# V-RBNN Based Small Drone Detection in Augmented Datasets for 3D LADAR System

**DOI:** 10.3390/s18113825

**Published:** 2018-11-08

**Authors:** Byeong Hak Kim, Danish Khan, Ciril Bohak, Wonju Choi, Hyun Jeong Lee, Min Young Kim

**Affiliations:** 1 School of Electronics Engineering, Kyungpook National University, Daegu 41566, Korea; byeonghak81.kim@hanwha.com (B.H.K); danish@ee.knu.ac.kr (D.K); 2 Hanwha Systems Corporation, Optronics Team, Gumi 39376, Korea; wonju09.choi@hanwha.com; 3 Faculty of Computer and Information Science, University of Ljubljana, SI-1000 Ljubljana, Slovenia; ciril.bohak@fri.uni-lj.si; 4 Agency for Defense Development, Yuseong, Daejeon 34186, Korea; hyunee@add.re.kr; 5 Research Center for Neurosurgical Robotic System, Kyungpook National University, Daegu 41566, Korea

**Keywords:** drone detection, clustering, 3D sensor, LiDAR, fusion data, 3D LADAR

## Abstract

A common countermeasure to detect threatening drones is the electro-optical infrared (EO/IR) system. However, its performance is drastically reduced in conditions of complex background, saturation and light reflection. 3D laser sensor LiDAR is used to overcome the problems of 2D sensors like EO/IR, but it is not enough to detect small drones at a very long distance because of low laser energy and resolution. To solve this problem, A 3D LADAR sensor is under development. In this work, we study the detection methodology adequate to the LADAR sensor which can detect small drones at up to 2 km. First, a data augmentation method is proposed to generate a virtual target considering the laser beam and scanning characteristics, and to augment it with the actual LADAR sensor data for various kinds of tests before full hardware system developed. Second, a detection algorithm is proposed to detect drones using voxel-based background subtraction and variable radially bounded nearest neighbor (V-RBNN) method. The results show that 0.2 m L2 distance and 60% expected average overlap (EAO) indexes are satisfied for the required specification to detect 0.3 m size of small drones.

## 1. Introduction

The increasing use of compact version of drones in the military, domestic and commercial sectors has raised a lot of privacy and security concerns. As of now, the development of countermeasures for potential drone threats is of great significance. Detecting the drone in the airspace is the first step of defense against them [1,2,3]. The electro-optical/infrared (EO/IR) systems based on the 2D image are efficient for detecting drones in both day and night time but they cannot differentiate between the background and the target cluster when images contain a complex background [4]. Additionally, such systems suffer from thermal image saturation due to which target sometimes overlap with the saturation region limiting the efficiency of the detection [5]. LiDAR is 3D sensor commonly used for outdoor target detection. In contrast to cameras, they provide accurate range information with a larger field of view. LiDAR is widely applied in autonomous vehicle systems and used as a countermeasure to avoid collision with pedestrians or other vehicles on the road [6,7,8,9]. They are by far the most used sensors for simultaneous localization and mapping (SLAM) which enable the robots to safely navigate in the unknown or GPS restricted environment and assists them in performing different complex tasks [10]. The working range of most LiDAR sensors is 100 m. Considering the drone interception and neutralization, this distance is restrained. The more practical approach is detecting the potential threat at about 1 to 2 km which allows enough time for the corresponding system to intercept the drone at the safe distance. To overcome this problem, the laser-based radar system (LADAR) to detect vehicles hundreds of meters away has already been developed [11,12,13]. We aim to develop a new LADAR system with high power, high response, and high resolution to effectively detect the approaching drones at a distance of 2 km. The manufacturing of optical components such as laser source and optics take a long time. Therefore, a long period of time is required to develop such a system. It is very challenging to design a robust and accurate threat detection system when the data from the real sensor is not available.

In this study, we present a technique for data augmentation with existing LiDAR and LADAR sensors for the development and testing of detection framework. A target is generated mimicking the anticipated behavior of the approaching drone in the range of 2 km. The shape, size, and trajectory of the target is simulated considering the optical design of the developing LADAR system [14,15,16]. Taking  into account that the LADAR system is not free from the optical and sensor noise, possible  noises are also included in the data. There are two advantages of designing a drone detection algorithm using augmented data. First, the detection software can be developed in parallel with the hardware. This reduces the overall production time and saves from the hassle of acquiring the data repeatedly from the real sensor as the hardware is modified multiple times during the development process. The augmented data allows the experimentation of various scenarios conveniently and makes the optimization much easier. Second, the ground truth of the target is accurate, and it is straightforward to measure the performance of the detection algorithm. Data recorded in the real operating environment should be visually checked by the trajectory of the target and the developer should make the reference data or mount the high precision RTK GPS sensor on the target and synchronize with the recorded data exactly. This process causes errors and degrades the reliability of the reference data.

**Figure 1:**
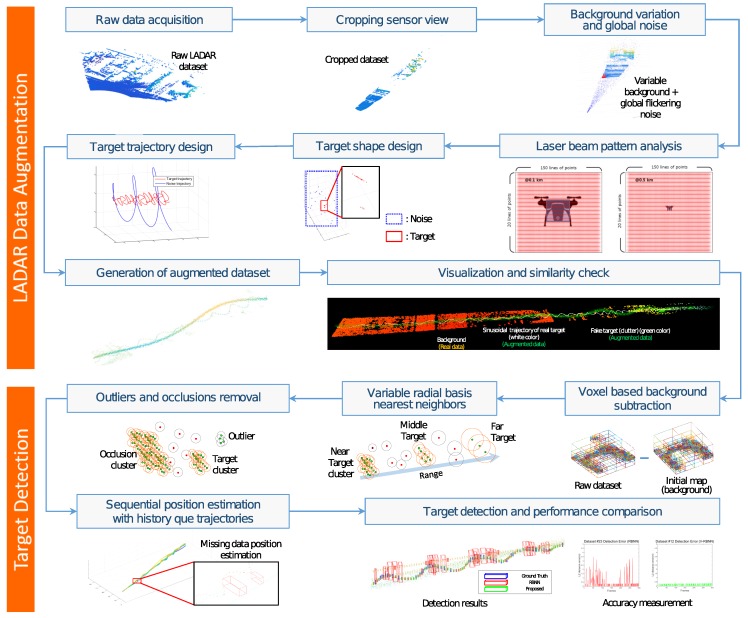
A flow chart for the small drone detection system.

Figure 1 shows the overall concept of generating the augmented dataset for various scenarios and designing the detection algorithm using these datasets. In the LADAR data augmentation step, the raw data is acquired by an existing LADAR sensor. The LADAR data is then cropped to get the field of view (FOV) of the developing LADAR sensor. On cropped data, the variational and general blinking noises are added in the background to simulate the effects of clouds and moisture. The time of flight (TOF) sensors, such as LADAR exhibit different point detection characteristics according to beam pattern and divergence angle. Therefore, the number of 3D points and the shape of the target is generated based on the distance of the target analyzing the laser beam characteristics. Next, a trajectory is designed keeping in view the movement of target, clouds, and other moving objects. Finally, the augmented dataset is generated by fusing the designed target shape and trajectory profiles. A visualization tool is used to visualize the augmented data in different colors and it is verified that the dataset exhibits similar nature as the actual situation. The augmented datasets are classified according to different scenarios and a bounding box is added to the absolute target location to calculate the ground truth data. In the target detection step, the detection algorithm can be developed using data designed in the LADAR data augmentation process. The initial map of the location is acquired beforehand. The moving objects and the static scene in the augmented data are separated using the octree-based comparison between the initial and other consecutive frames of the map [17]. Next, the candidate targets are classified by the radially bounded nearest neighbor (RBNN) clustering method [18]. As the characteristics of the target vary with the distance, A new variant of RBNN is proposed in this work which uses variable radius values instead of a single predefined radius to cluster the data. We call this method variable radially bounded nearest neighbor (V-RBNN) clustering. The clustering results can suffer from noise and interference of the objects close to the target. To tackle this issue, clusters are further processed with an outlier removal technique based on minimum points in the radius constraint using nearest neighbor search. During the experimentation, we observed that sometimes the target is not detected, or the detected bounding box is extraordinarily large due to outliers in the augmented datasets with higher noise. To overcome such a situation, sequential target detection is monitored, and a queue of the detection result is maintained to predict the failure. Finally, in the quantitative measurement section, the L2 distance of the center coordinates and the intersection over union (IOU) of the two bounding boxes are compared with the ground truth and the conventional RBNN method to measure the performance of the target detection algorithm. The performance of the overall algorithm can be compared with the average Euclidean (L2) distance and expected average overlap (EAO) values for different scenarios (datasets).

## 2. Related Works

### 2.1. EO/IR Imaging System for Drone Detection

**Figure 2:**
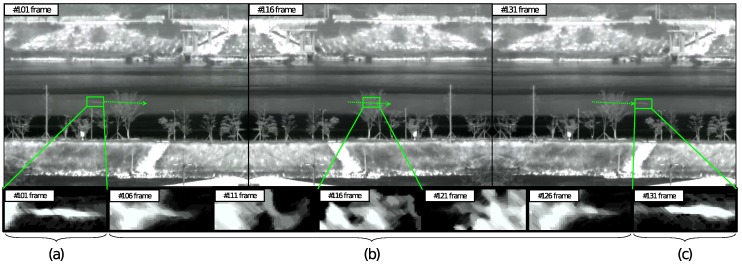
Drone detection using EO/IR imaging system at different background condition. (**a**,**c**) detection success in normal background, (**b**) detection fail in complex background.

EO/IR-based systems are widely used for the detection of potential threats [19,20]. Compared to the normal imaging sensor, EO/IR can detect the target even in the darkness of night. Figure 2 shows the EO/IR images of the drones approaching in a transverse sinusoidal direction at a distance of about 1000 m. There are mountains and roads in the distant background along with a river in the middle of the images. There are trees on the river banks at near range, and a drone is flying in front of the trees. As shown in Figure 2a,c, the drone target is optimally detected where the background is clear. However, it can be seen in Figure 2b the detection fails when the drone approaches the region containing the tree in the background. These conditions frequently occur in the EO/IR operating environment due to the presence of trees, mountains, and clouds in the scene or because of the reflection of the sea surface, sunglint phenomenon or flame of the firing cannon.

### 2.2. Basic Experiment Using 3D LiDAR

**Figure 3:**
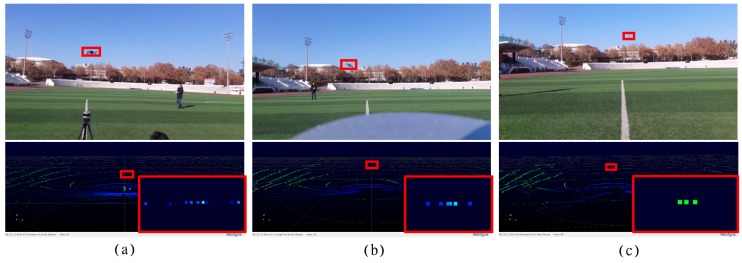
Limitation of target detection using 3D LiDAR sensor system. (**a**) Range = 10 m, 9 points detection, (**b**) Range = 25 m, 6 points detection, (**c**) Range = 50 m, 3 points detection.

3D sensors can be used to overcome the limitations of EO/IR sensors in drone detection [21]. Figure 3 shows the detection result of a small sized drone of 30cm${}^{3}$ using a LiDAR sensor (VLP-16) at different distances. The Angle resolution of the LiDAR used in the experiment is 0.1${}^{\circ }$ in the horizontal direction and 2.0${}^{\circ }$ in the vertical direction. The detection resolution of the drone calculated from the tangent function using Equations (1) and (2) is 0.017 m (1.7 cm) in the horizontal direction and 0.3492 m (34.9 cm) in the vertical direction at distance 10 m.

(1) $${AZ}_{RES}=\tan\left({AZ}_{angle}\times \frac{PI}{180}\right)\times Range$$

(2) $${EL}_{RES}=\tan\left({EL}_{angle}\times \frac{PI}{180}\right)\times Range$$

(3) $$AZ\_points=\frac{Target\_size}{{AZ}_{RES}},EL\_points=\frac{Target\_size}{{EL}_{RES}}$$

where, AZ is azimuth and EL is the elevation. ${AZ}_{RES}$ is AZ  resolutionand ${EL}_{RES}$ is EL resolution.

The scanning resolution of a 30cm${}^{3}$ drone calculated for 10 m using Equation (3) is 17 points (maximum) in the horizontal direction and 1 point in the vertical direction. However, Figure 3a shows that at the distance of 10 m from the LiDAR sensor, the horizontal resolution of the drone is 9 points and the vertical resolution is 1 point. Similarly, it can be seen in Figure 3b the horizontal resolution is 6 points and vertical resolution is 1 point at the distance of 25 m. Figure 3c shows the test result at 50 m. According to the formula the laser scanning resolutions, ${AZ}_{RES}$ and ${EL}_{RES}$ are 0.0873 m and 1.746 m respectively and the small sized target is represented as 3.4×0.17 points, but the actual detected points are 3×1. The resolution reduces as the drone moves farther away from the LiDAR. At 2 km, the ${AZ}_{RES}$ and ${EL}_{RES}$ of the LiDAR are 3.49 m and 69.84 m and the detectable points are 0.086×0.0043. Hence only one point is predicted with intermittent blinking with low probability. Considering the blinking noise reflected by the moisture in the air, the system cannot distinguish between the noise and target drone at this distance. Thus, the LiDAR sensor has a very low resolution and it is not suitable for detecting drones at long distance.

### 2.3. Proposed 3D Scanning System (LADAR)

**Figure 4:**
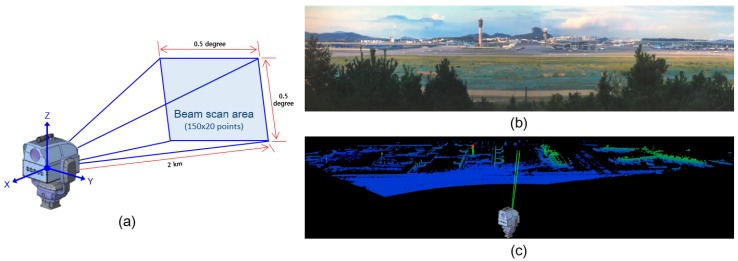
LADAR concept and the example dataset of an airport. (**a**) LADAR hardware design and scanning view, (**b**) experimental environment (airport), (**c**) an example sensing result.

To tackle the aforementioned issues of 2D EO/IR imaging sensor and 3D LiDAR sensor in drone detection, we develop a LADAR system as shown in Figure 4a. The sensor is able to detect high-resolution points within $0.5^{\circ }\times 0.5^{\circ }$ by scanning the high-resolution laser pulses at high speed in AZ and EL directions. Figure 4b shows the initial map experimental environment using a prototype model, Figure 4c shows the result of the initial map acquisition, the green guideline denotes the real-time scan view of LADAR. Once the initial map has been generated, LADAR refers to the radar signal and directs it to the approximate position, then a laser starts scanning to detect the target. A wide range of scanning can be performed by rotating the scanner assembly using two-axis gimbal equipped with a servo motor. A 1560 nm laser source with a 1 *n*sec pulse duration is used to achieve the high resolution. For high-speed scanning, 1 kHz fast mirror galvanometer is used. The pixel size of the light receiving detector unit is 100 μm, and the optical system is added to the laser detector unit. The arrival beam diameter is designed for a footprint of 100 μm or less. Considering beam divergence and galvanometer scanning characteristics, the scannable laser beam array is 150×20. Therefore, a $0.5^{\circ }\times 0.5^{\circ }$ space (angular) resolution is calculated as $0.025^{\circ }\times 0.003^{\circ }$ in the local scan FOV space. Compared to the conventional LiDAR sensors, this performance is 80 times better in the EL direction and 33 times in the AZ direction. LADAR scans the local optical scanning space with a servo motor driver to scan 350${}^{\circ }$ in the AZ direction and 120${}^{\circ }$ in the EL direction. The maximum detectable distance is 2 km, which is 16 times higher than the conventional LiDAR sensor. To detect a target at a distance of 2 km, a laser output of 700 kW pulse peak power and a seed light-based optical fiber amplifying laser with a fast repetition rate of 1000 kHz is used.

### 2.4. Detection Speed of LADAR

The maximum speed of the detectable drones is limited by the optical scanning speed, the speed of two-axis gimbal motor, and the range between the sensor and the drones. The optical scanning speed of LADAR is up to 20 Hz and the mechanical motor speed is up to $16^{\circ }$/s. The maximum speed of the detectable drones is about 56 m/s (202 km/h) at 200 m. If the distance is 100 m, the maximum detectable drone speed is 28 m/s (101 km/h). The optical scanning speed should be fast, in the case of fast direction switching and zigzag movement, the trajectory information of at least two center coordinates of drones maintain accuracy. At a distance of 200 m, 10 m/s (36 km/h) dynamic flying drones are detected at 3 center coordinates in 20 Hz optical scan condition and 17 center coordinates in 1000 m range.

## 3. Generation of Targets and Noises

Designing a high-performance LADAR is a long-term project and design changes are inevitable during the implementation phase. Therefore, it is almost impossible to conduct experiments for acquiring laser data frequently during the hardware development. Also, if the implementation of the detection algorithm has started after the hardware production is completed, the entire development period will be very slow. It is also difficult to obtain various reference datasets by performing target detection experiments using actual LADAR. In this section, we analyze the beam characteristics of the laser sensor using the previously acquired reference map data and describe the shape of the target. The augmented data generated in this procedure is used in the process of developing the target detection algorithm.

### 3.1. Laser Beam Analysis

Accurate analysis of laser beam is a critical step to model the augmented data for LADAR sensor. The number of 3D points of the target can be predicted by calculating the interval and number of laser pulses detected in the FOV considering the aforementioned beam divergence angle and the scanning resolution of LADAR.

**Figure 5:**
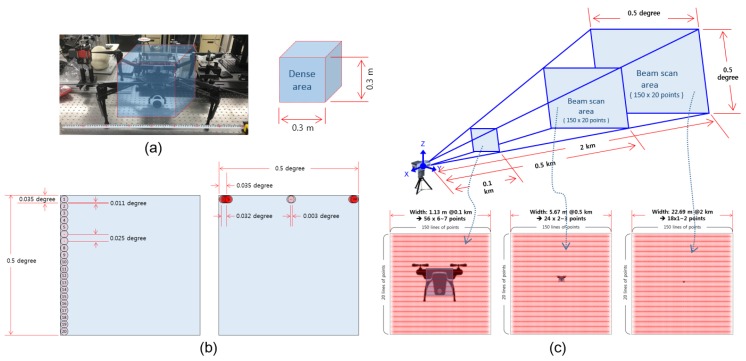
Characteristics analysis of LADAR laser beam and calculation for equivalent target projection points. (**a**) Dense area of target, (**b**) beam divergence and intersection, (**c**) detectable target projection points.

Figure 5a shows the shape of a small drone with a volume of 30 cm${}^{3}$ as a detected target and its dense region. Figure 5b illustrates the intersection of the vertical and horizontal direction of the laser beam. The laser beam interval and the angle with respect to the crossing region can be seen in the same figure. The intersection angle of the vertical direction (EL direction) is 0.003${}^{\circ }$, and the intersection angle of horizontal (AZ direction) is 0.011${}^{\circ }$. When the FOV in the AZ direction and the EL direction is 0.5${}^{\circ }$, the length (m) of the FOV is changed by the distance and can be simply obtained by Equation (4). If the scan resolution in the AZ direction is set to $SR_{AZ}=150$ and $SR_{EL}=20$ in the EL direction, the number of lines occupied by the target can be obtained using Equation (5) where, $T_{m}$ is length (m) of the target and ROUNDUP is a function to derive integer values. If the beam divergence angle is designed to be $B_{deg}=0.62\;mrad$ (0.035${}^{\circ }$), the length information $\left(BI_{AZ},BI_{EL}\right)$ for the intersection area of the beam can be calculated by considering $SR_{AZ}$ and $AR_{EL}$ using Equation (6). The length considering the detection area added to the beam intersection area can be calculated as in Equation (7), and the number of 3D points in the final target can be calculated by Equation (8).

(4) $${FOV}_{m}=2\times \tan\left(FOV\times \frac{PI}{180}\times 0.5\right)\times R_{m}$$

(5) $${BL}_{AZ}=ROUNDUP\left(\frac{T_{m}}{{FOV}_{m}/{SR}_{AZ}}\right),{BL}_{EL}=ROUNDUP\left(\frac{T_{m}}{{FOV}_{m}/{SR}_{EL}}\right)$$

(6) $${BI}_{AZ}=\left(B_{deg}\times {SR}_{AZ}\right)-{FOV}_{deg}\left({SR}_{AZ}-1\right),{BI}_{EL}=\left(B_{deg}\times {SR}_{EL}\right)-{FOV}_{deg}\left({SR}_{EL}-1\right)$$

(7) $${TBI}_{AZ}=\left(T_{m}+{BI}_{AZm}\right)\times 2,{TBI}_{EL}=\left(T_{m}+{BI}_{ELm}\right)\times 2$$

(8) $$TP=ROUNDUP({TBI}_{AZ}\times \left(\frac{{SR}_{AZ}}{{FOV}_{m}}\right)\times {TBI}_{EL}\times \left(\frac{{SR}_{EL}}{{FOV}_{m}}\right))$$

Table 1 shows the results of the aforementioned calculations with respect to the distance. In the case of 200 m, it is predicted that the target is detected by 20 × 3 lines and represented by 105 3D points by adding an additional point for crossing region of the beam. Consequently, the target will be detected by 2 × 1 lines at 2 km and the result reflecting the beam crossing area is represented by 17 points. The LADAR can represent 17 AZ and 1 EL points compared to the LiDAR sensor which represents 0.086 AZ and 0.0043 EL points at the distance of 2 km. Figure 5c shows the result of projecting the point characteristics of the final detected target.

### 3.2. Shape of Target and Noise

**Figure 6:**
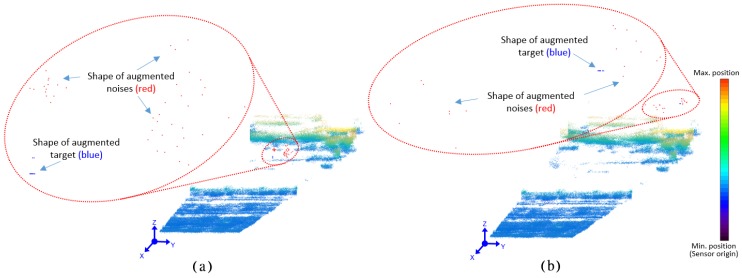
Shape of targets and noises at different ranges. (**a**) target shape at middle range, (**b**) target shape at far range.

To generate the target, first the number of 3D points contain by the target is calculated, then the background shape is created according to the number of 3D points. Finally, the generated shape of the target is verified. Figure 6a shows the result of designing the shape of the target at the middle distance of about 500 m along with other background (disturbance) elements. Figure 6b shows the result at a distance of 1 km or more. Since the shape of the target is randomly distributed during the experiment using LiDAR, the characteristics of the random distribution are added in consideration of the size of the target and the number of points by the distance. Therefore, different profiles are created for different datasets.

### 3.3. Trajectory Design

**Figure 7:**
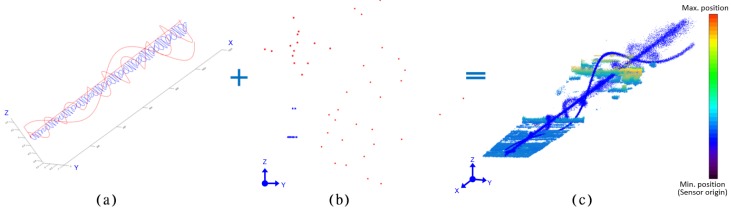
Trajectory design for targets and noises. (**a**) trajectory design, (**b**) target shape design, (**c**) generation of augmented experimental dataset.

The datasets for the experiments are designed assuming different trajectories of the target. Different datasets are created by first keeping the same shape of the target with variation in trajectories and then by varying the shape of targets keeping the same trajectory. The blue line in Figure 7a is the trajectory plan for the target movement and the red color shows the result of the generation of the motion characteristics of different fake targets. The lower frequency of the trajectory of the fake target simulates a slowly moving object such as a cloud, and the red line moving in a linear pattern simulates the noise distributed near the target. Figure 7b shows the shape of the target corresponding to a frame. Blue dots represented by two lines correspond to the target, and red dots denote various types of noise. Figure 7c shows a data set that is fused to generate the trajectory profile with target information and background information. The part represented by jet color map is the background, and the part shown in blue is the data with the target and added noise.

## 4. Augmentation and Visualization

**Figure 8:**
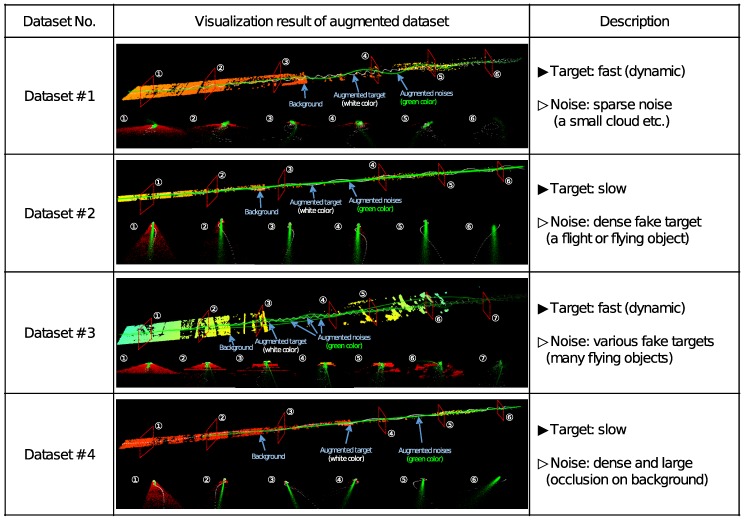
Numbers of visualization result of augmented datasets and its descriptions.

The success of the detection algorithm depends on the augmented datasets generated using the background data. It is necessary to verify if the generated datasets meet the desired requirements. It should be confirmed that the shape and movement of the target are appropriate, and interference of the noise is similar to the imitated situations. To examine the datasets, we designed a web-based visualization tool to visualize and verify the augmented data classified into four datasets regarding the speed of the target and the amount of noise in the data. Figure 8 shows the visualization result and the description of each dataset. Dataset #1 contains the profile of the fast-moving drone target which moves in a winding path and sparse random 3D points to simulate the interference of small clouds and sensor noise. The speed of the target is slow in dataset #2 and dense 3D points are added close to the target to create the effect of birds and other flying objects around the target. Dataset #3 is generated to test the performance of the detection algorithm for fast-moving targets with dense or coarse noise that interfere with one another. In dataset #4, the interference of large planes, thick clouds, and high buildings is imitated with the slow-moving target. The generated datasets after the assessment in the visualization tool can be used for the algorithm development.

## 5. Target Detection

The overall process of designing a small-sized drone target detection algorithm using augmented data for the experiment is shown in Figure 1. Algorithm 1 describes the algorithm for target detection that works as follows. First, in the background subtraction stage, we use Background_pointXYZ and Current_pointXYZ to compare the initial map with input data to segment out the same static objects in the scene. The output of this operation is the cloud_Octree (further described in Section 5.1). In the V-RBNN step, we estimate the distance from the target based on the history of detection and store it as $calRange_{t}$. radius is obtained by taking the product of $h_{scan}$, the number of horizontal scan lines, and $v_{scan}$, the number of vertical scan lines, and the length of the LADAR FOV, using tan() function, with respect to ${calRange}_{t}$. At this point, the λ and bias constant parameters can be adjusted depending on the detection target size. After clustering, the outliers are removed with the minimum number of neighbor points, mMin within a predefined radius. In the experimental results, mMin is set to 3. To overcome the interference that occurs when large clusters other than the target come into the sensing area after the outliers have been removed, we check the size of the target based on the diagonal length of the detected bounding box. In case of abnormally large diagonal condition diagBB > 1 m, the relevant detected bounding box is excluded. Finally, there are rare occasions when no target points are found, or abnormal detection results are obtained. In this case, an exceptional situation is detected, and the current target location is predicted using the history of previous target detection based on a finite impulse response (FIR) filtering method [22].

**Algorithm 1:** The V-RBNN-based target detection.
1:  // Background subtraction

2:        PointXYZ::cloud_Base=Background_pointXYZ

3:        PointXYZ::cloud_Cur=Current_pointXYZ

4:        cloud_Octree←OctreeChangeDetector(PointXYZ)

5:  // V-RBNN

6:        ${calRange}_{t}\leftarrow TargetRangeCal\left(history\_que\_cenBB\_xyz\left[i\right]\right)$

7:        λ=7.0,bias=0.01   (//tunable constant parameters)

8:        $radius=\lambda \;/\;\left(h_{scan}\times v_{scan}\right)\times \tan\left(FOV\times \left(\pi /180\right)\right)\times {calRange}_{t}\;+\;bias$

9:        cloud_cluster←SetRBNNRadius(cloud_Octree,radius)

10: // Outlier and occlusion removal

11:      Outlier_remove←setMinNeighborsInRadius(cloud_cluster,nMin)

12:      **if** ( diagBB>1 )

13:         Occlusion_remove=Outlier_remove−maxDiagBB

14:      **else**

15:         cloud_cluster_target=Outlier_remove

16: // Sequential position estimation

17:      **if** ( ${calRange}_{t}\;<\;1\;\left|\right|\;\left({calRange}_{t}\;-\;{calRange}_{t-1}\right)\;>\;1000$ )

18:         Final_target_BB←estimateBB(history_que_cenBB_xyz[i])

19:      **else**

20:         Final_target_BB←drawBB(cloud_cluster_target)


### 5.1. 3D Background Subtraction

When the initial 3D map of an environment is generated, the spatial change can be realized by comparing it with new arriving frames in real time [9,23]. We used the octree-based occupancy grid representation method [24] to distinguish between the constant part of the scene and the moving target. The octree is a kind of tree data structure in which each node has eight child nodes. The octree is widely adopted to partition the 3D spaces because it is computationally efficient for such data [25,26,27]. The voxel-based octree structure of the two point clouds (initial or reference map and the receiving frames) are compared and the resulting point cloud is the moving target with a lot of clutter. This point cloud is then processed to differentiate between the target and the noise.

### 5.2. Variable Radially Bounded Nearest Neighbor (V-RBNN)

**Figure 9:**
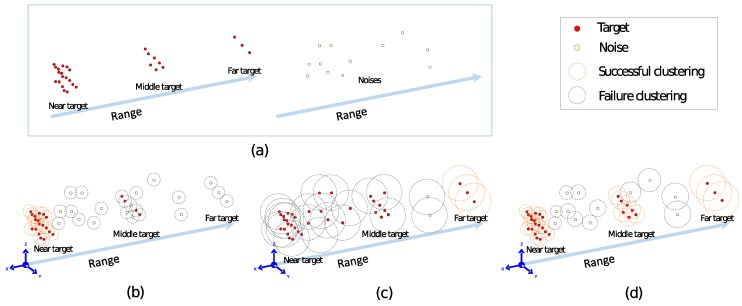
Basic concept of variable radial basis nearest neighbor clustering method. (**a**) Ground truth, (**b**) RBNN (R = 0.06 m), (**c**) RBNN (R = 0.2 m), (**d**) Proposed.

There are several approaches for clustering the data in 3D space [28,29,30]. A representative approach is the nearest neighbor search, we employed a radially bounded nearest neighbor graph method (RBNN) [18]. This is the modified form of nearest neighbor graph method in which every node is connected to all the neighbors present in a predefined radius. The RBNN is fast and can be used to cluster the data in real time because for every node the nearest neighbor query is not required eliminating the need for rearranging the graphs. However, the original RBNN cannot be applied for the clustering in long-range sensors. Due to the varying nature of shape and size of the target acquired by the LADAR sensor, a fixed radius cannot be defined for all the distances. The target has a dense shape and a large number of 3D points when it is closer to the sensor. Clustering with the smaller radius in such case will result in the no detection (failure) or the detection of noise as a target cluster. Conversely, the large radius will cause the detection of outliers with the target when it is approaching from a long distance. To effectively cluster the targets ranging from 1 to 2 km, a variable radially bounded nearest neighbor (V-RBNN) method is proposed considering the distribution of the targets varying by the approaching distance. Figure 9a shows the distribution of the shape of the near, medium, and long distance target points and the noise as a reference. Figure 9b shows that when the length of the search radius is set to 0.06 m, the performance for the medium-range remote target results in failure. If the radius is set to 0.2 m as shown in Figure 9c, the detection performance deteriorates. We adjusted the radius value adaptively according to the distance of the target as shown in Figure 9d so that clustering performance is maintained even if the target point distribution is varied. The green dot is the part of the target to be judged, and the red is the noise (clutter). An orange circle indicates the optimal clustering of the target, and a gray circle denotes the clustering failures, or a non-target noise detected as a target.

### 5.3. GUI Software and Experiments

**Figure 10:**
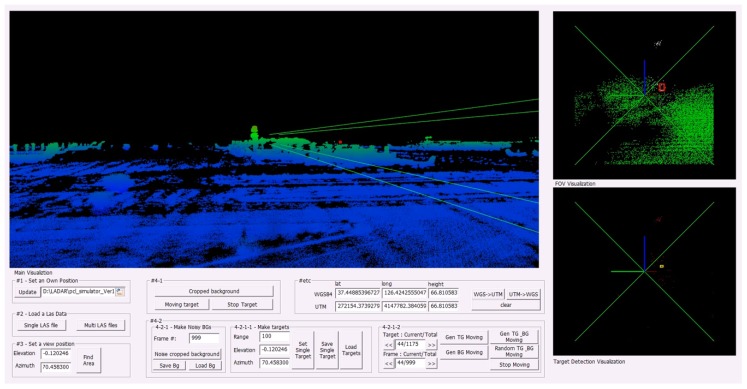
GUI software environment for small target detection.

**Figure 11:**
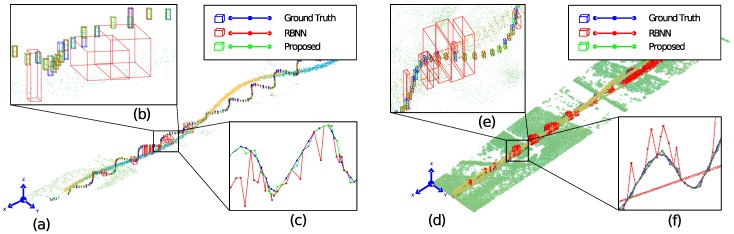
Experimental results of small drone detection. (**a**) detection result using dataset #1, (**b**) bounding box results for each frames, (**c**) Euclidean distances between sequential targets, (**d**) detection result using dataset #3, (**e**,**f**) show bounding box and Euclidean.

For the implementation and testing of the target detection algorithm, a graphical user interface (GUI) software is designed as shown in Figure 10. The GUI reads the augmented data displayed in the large window on the upper left where green lines express the FOV of LADAR. The motion of the reference target is marked as a red bounding box. Below the big windows, data loading button and parameter input boxes to adjust the camera view are located. The small window on the upper right shows the area of the FOV detected by LADAR in real time and the movement of the reference target in a red color bounding box. The small window on the bottom right shows the final target detection area as a yellow bounding box. We verified the results of the bounding box detection of the right bottom windows (above: ground truth, below: detection result) in the final implemented GUI experimental environment. Figure 11a is the #1 experimental dataset identified in Figure 8a and Figure 11d is the #1 experimental dataset identified in Figure 8c. Figure 11b,e shows the baseline target, the existing RBNN method, and the BB detection result of the proposed method in each dataset. Figure 11c,f are the L2 distances between the target center coordinates of the proposed method. In Figure 11b,e, the existing RBNN method detects the target including fake targets when they are close and detects BB abnormally. Therefore, the L2 distance of the center coordinate detection result of the target becomes larger. The proposed method shows that this problem can be solved.

## 6. Quantitative Measurement

**Figure 12:**
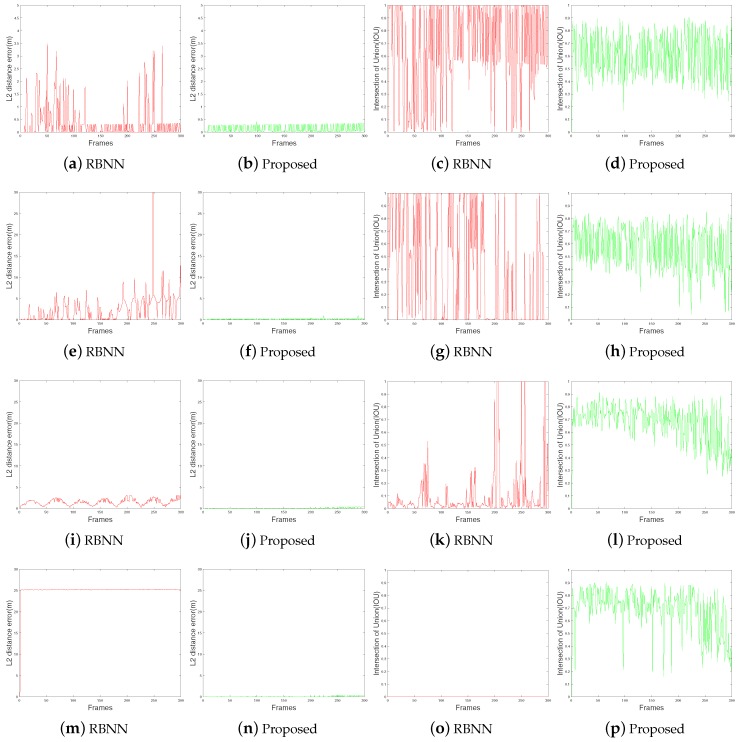
Euclidian distance and intersection over union (IOU) measurement. (**a**–**d**) result using dataset #1, (**e**–**h**) result using dataset #3, (**i**–**l**) result using dataset #2, (**m**–**p**) result using dataset #4.

**Figure 13:**
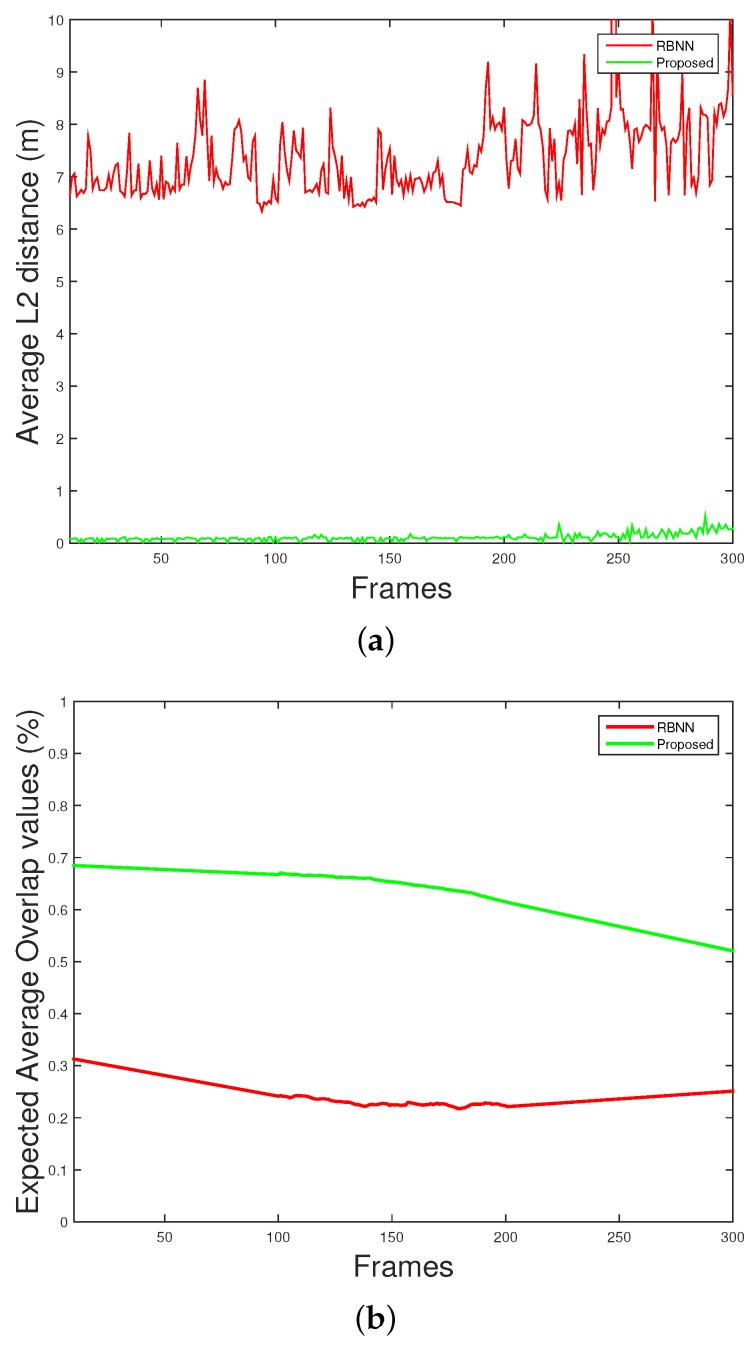
Overall performance measurement (**a**) Average L2, (**b**) expected average overlap (EAO).

It is important to quantitatively evaluate the performance of the target detection algorithm. The GUI software used in the experiments was designed to save log files for target detection results. There are two indexes used for quantitative measurements. First, the error between the position of the reference target and the center coordinates of the target position of the detection result was measured as L2 distance. Figure 12a,e,i,m shows the L2 distance of the target detection result using the conventional RBNN method. Figure 12b,f,j,n shows the results for the proposed method. Compared with the conventional RBNN method, the error is significantly lower and stable characteristics are shown. The second index is the IOU. This shows how the target detection bounding box differs from the reference area. The L2 distance is a good property when the response value is small, whereas the IOU is good when the value is close to 1. Figure 12c,g,k,o shows the EAO (Estimated Average Overlap) measurement results for the target detection using the conventional RBNN method. Figure 12d,h,l,p shows the results for the proposed method. Figure 12 shows that the proposed algorithm is superior in performance, but the IOU measurement data becomes wider because of the increase and decrease of each frame. In this case, the average algorithm performance can be evaluated by measuring the EAO. Figure 13a shows the average L2 distance. Figure 13b shows that the accuracy of the V-RBNN is 0.6 to 0.7 which is almost 2X better than the conventional RBNN method.

## 7. Conclusions

The first contribution of this paper is that we presented a data augmentation method for the design and testing of drone detection algorithm without real sensor data. The proposed method can be employed to simulate output of any LADAR sensor by changing the sensor characteristics to achieve the desired shape and size of the drones and the resolution of 3D points can be adjusted accordingly. A diverse set of data can be generated for the experimentation and design of the detection framework. The implementation of long-range detection algorithm using a LADAR sensor model and augmented dataset for small drones is the second contribution of this paper. The given method can detect drones at the range of almost 2 km. A new clustering method (V-RBNN) is investigated as the conventional clustering method (RBNN) is not effective to classify the target due to variation in drone shape and size at the different distances. Results demonstrates that the V-RBNN substantially improve the accuracy of the clustering. We expect that this study will be contributed to development of detection algorithms for 3D sensor systems in various applications. Also, it can be widely used as a technique to quickly optimize its software before hardware development is completed.

The distinction between birds and drones is a challenging task in small drone detection system. This issue is in our research pipeline and in the future, we will acquire datasets including birds and drones after the development of actual LADAR system. We consider probability-based intelligent classification methods using analysis of flight behavior of birds and drones.

## Figures and Tables

**Table 1:** Calculation for target projection points in different ranges.

Range	FOV	Target Angle	AZ Lines	EL Lines	AZ + BI	EL + BI	Target
(R_m)	(FOV_m)	(T_deg)	(BL_AZ)	(BL_EL)	(TBI_AZ)	(TBI_EL)	(TP)
200	2.27	0.086	20	3	0.519	0.322	105
500	5.67	0.034	8	2	0.848	0.355	46
800	9.08	0.021	5	1	1.177	0.389	20
1100	12.48	0.016	4	1	1.506	0.422	19
1400	15.88	0.012	3	1	1.834	0.455	18
1700	19.29	0.010	3	1	2.163	0.489	17
2000	22.69	0.009	2	1	2.492	0.522	17
